# Supplementary figures and images for: Silver-spoon effect in agricultural crop consumers: crop consumption enhances skeletal growth in sika deer
Source: PeerJ. 2025 Aug 7;13:e19836. doi: 10.7717/peerj.19836 (PMC12335832; doi:10.7717/peerj.19836)

Model 3 ( $\Delta AIC=1.87$ )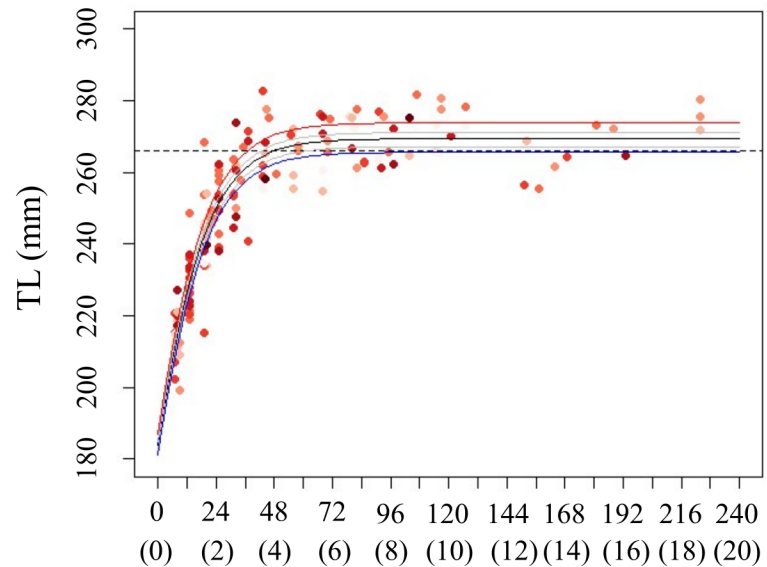Model 1 ( $\Delta AIC=1.89$ )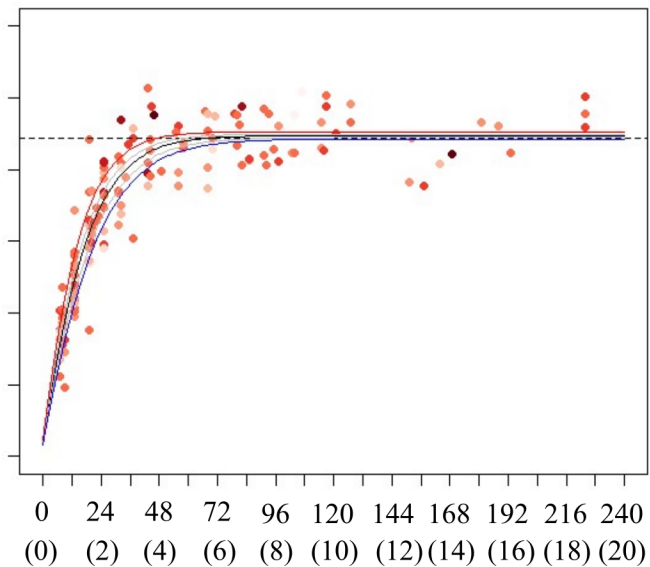Model 4 ( $\Delta AIC=3.21$ )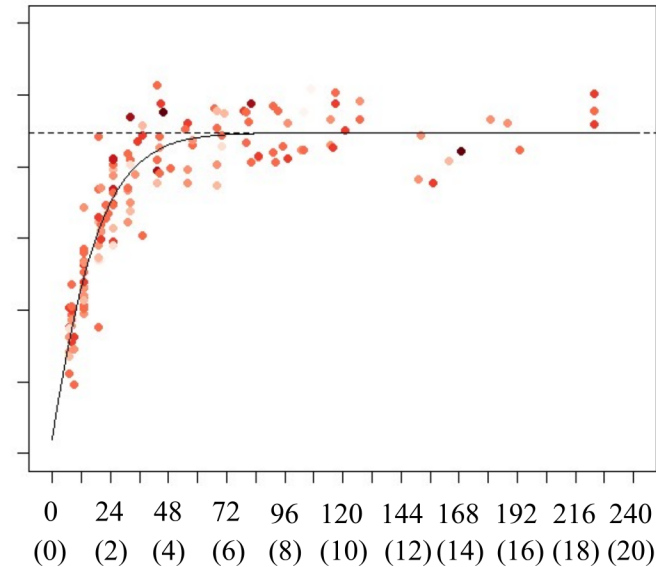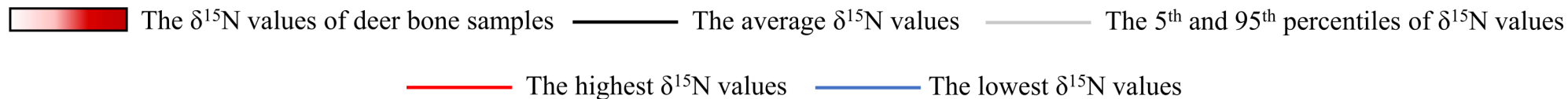

Supplement: Supplemental Information 1 — Models were shown in order of ΔAIC. Grey curves were estimated by the model that incorporated the 5th (1.0‰) and 95th (4.9‰) percentiles of the δ15N values of bone samples as a parameter of the growth curve. Blue and red curves were estimated by the model that incorporated the lowest (–0.5‰) and the highest (7.5‰) δ15 N values of the bone samples as a parameter of the growth curve, respectively. Numbers in parentheses on the horizonal axis indicate the age in years. The data points represent individual deer, with darker red indicating higher δ15N values. The horizonal dashed line indicates the asymptotic value estimated by the best model. [file peerj-13-19836-s001.pdf]

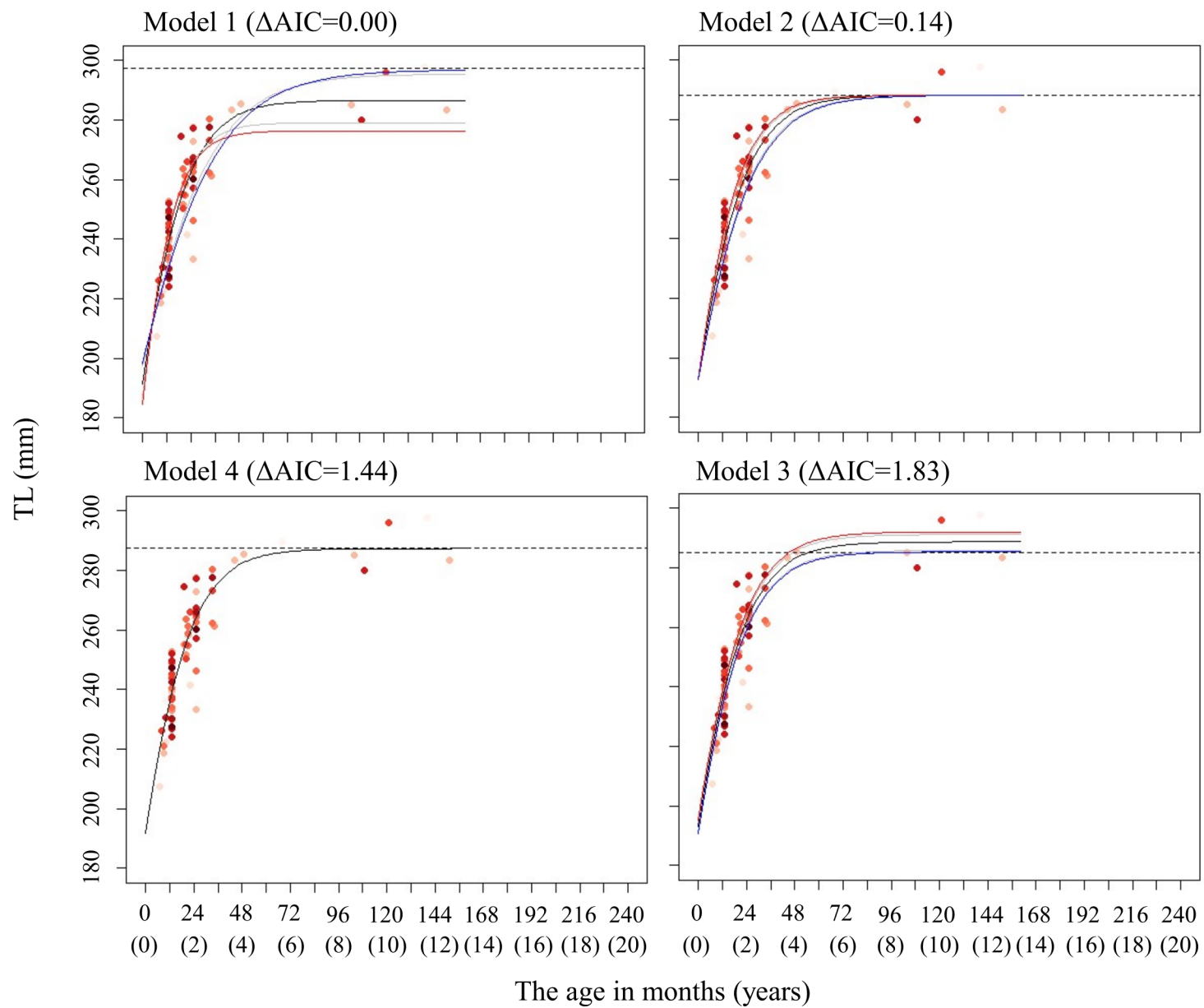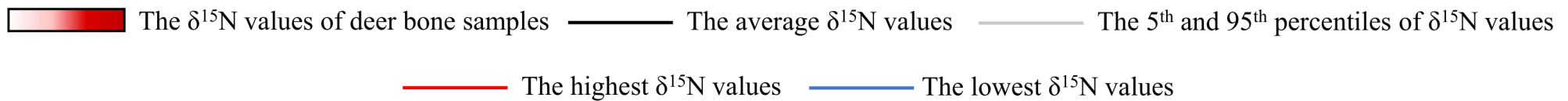

Supplement: Supplemental Information 2 — Models were shown in order of Δ AIC. Grey curves were estimated by the model that incorporated the 5th (0.5‰) and 95th (4.6‰) percentiles of the δ15N values of bone samples as a parameter of the growth curve. Blue and red curves were estimated by the model that incorporated the lowest (0.2‰) and the highest (5.3‰) δ15N values of the bone samples as a parameter of the growth curve, respectively. Numbers in parentheses on the horizonal axis indicate the age in years. The data points represent individual deer, with darker red indicating higher δ15N values. The horizonal dashed line indicates the asymptotic value estimated by the best model. [file peerj-13-19836-s002.pdf]
