# Supplementary material for: Silver-spoon effect in agricultural crop consumers: crop consumption enhances skeletal growth in sika deer
Source: PeerJ. 2025 Aug 7;13:e19836. doi: 10.7717/peerj.19836 (PMC12335832; doi:10.7717/peerj.19836)
Supplement: Supplemental Information 3 — The δ15N values were measured for the samples indicated in the column “bone sample type”. [file peerj-13-19836-s003.docx]

| ID | hunting date | sex | Total length of the skull (mm) | age in month | age in year | δ^15^N (‰) | bone sample type |
| --- | --- | --- | --- | --- | --- | --- | --- |
| GMT060 | 2018/1/12 | F | 255.55 | 43 | 3 | 2.0 | collagen |
| GMT075 | 2018/1/23 | F | 273.28 | 115 | 9 | 2.4 | collagen |
| GMT078 | 2018/1/26 | F | 264.78 | 103 | 8 | 2.9 | collagen |
| KO213 | 2018/1/26 | F | 273.31 | 127 | 10 | 3.4 | collagen |
| KO0127 | 2018/1/27 | F | 264.11 | 91 | 7 | 3.9 | collagen |
| KO215 | 2018/1/27 | F | 275.73 | 79 | 6 | 4.1 | collagen |
| GMT088 | 2018/1/28 | F | 265.88 | 115 | 9 | 2.5 | collagen |
| GMT089 | 2018/1/29 | F | 208.53 | 7 | 0 | 3.0 | collagen |
| GMT097 | 2018/1/30 | F | 244.63 | 19 | 1 | 1.7 | collagen |
| KO217 | 2018/2/4 | F | 218.79 | 8 | 0 | 3.8 | collagen |
| KO219 | 2018/2/5 | F | 272.18 | 56 | 4 | 4.3 | collagen |
| KARU193 | 2018/2/6 | F | 233.97 | 20 | 1 | 0.2 | collagen |
| KO220 | 2018/2/7 | F | 244.04 | 20 | 1 | 3.4 | collagen |
| KO221 | 2018/2/7 | F | 264.7 | 104 | 8 | 3.6 | collagen |
| GMT111 | 2018/2/9 | F | 241.73 | 20 | 1 | 2.9 | collagen |
| KO222 | 2018/2/9 | F | 259.18 | 56 | 4 | 2.9 | collagen |
| GMT112 | 2018/2/11 | F | 262.1 | 32 | 2 | 3.3 | collagen |
| KO223 | 2018/2/11 | F | 211.11 | 8 | 0 | 4.5 | collagen |
| GMT118 | 2018/2/12 | F | 268.86 | 152 | 12 | 2.7 | collagen |
| GMT123 | 2018/2/20 | F | 261.55 | 164 | 13 | 1.3 | collagen |
| SA005 | 2018/2/21 | F | 275.46 | 224 | 18 | 3.3 | collagen |
| GMT125 | 2018/2/23 | F | 272.22 | 188 | 15 | 3.0 | collagen |
| SA006 | 2018/2/23 | F | 280.44 | 224 | 18 | 4.1 | collagen |
| GMT148 | 2018/2/26 | F | 250.09 | 32 | 2 | 2.3 | collagen |
| SA008 | 2018/3/3 | F | 277.62 | 117 | 9 | 4.0 | collagen |
| KARU194 | 2018/3/31 | F | 247.18 | 21 | 1 | 0.1 | collagen |
| KO236 | 2018/3/31 | F | 280.6 | 117 | 9 | 3.5 | collagen |
| KO005 | 2018/4/6 | F | 275.27 | 46 | 3 | 7.3 | collagen |
| KO006 | 2018/4/6 | F | 277.61 | 82 | 6 | 6.0 | collagen |
| KO013 | 2018/4/15 | F | 249.34 | 22 | 1 | 3.5 | collagen |
| KO016 | 2018/4/16 | F | 258 | 34 | 2 | 2.7 | collagen |
| KARU003 | 2018/4/22 | F | 265.59 | 70 | 5 | 0.7 | collagen |
| KARU004 | 2018/4/25 | F | 266.04 | 58 | 4 | 3.1 | collagen |
| KARU007 | 2018/5/3 | F | 274.84 | 71 | 5 | 1.7 | collagen |
| KO026 | 2018/5/12 | F | 246.84 | 23 | 1 | 3.4 | collagen |
| KARU014 | 2018/5/14 | F | 265.66 | 95 | 7 | 3.4 | collagen |
| KO031 | 2018/5/15 | F | 267.17 | 35 | 2 | 4.0 | collagen |
| KARU023 | 2018/5/17 | F | 281.72 | 107 | 8 | -0.5 | collagen |
| KO19-220 | 2019/1/2 | F | 268.28 | 19 | 1 | 3.7 | collagen |
| KO19-221 | 2019/1/3 | F | 278.13 | 127 | 10 | 3.0 | collagen |
| GMT19-430 | 2019/1/12 | F | 263.73 | 31 | 2 | 3.6 | collagen |
| KO19-230 | 2019/1/13 | F | 253.8 | 19 | 1 | 3.6 | collagen |
| KO19-234 | 2019/1/14 | F | 277.06 | 91 | 7 | 3.5 | collagen |
| GMT19-435 | 2019/1/18 | F | 215.16 | 19 | 1 | 3.9 | collagen |
| GMT19-436 | 2019/1/18 | F | 233.92 | 19 | 1 | 0.7 | collagen |
| GMT19-446 | 2019/1/22 | F | 253.52 | 31 | 2 | 2.3 | collagen |
| KARU19-258 | 2019/1/23 | F | 270.39 | 55 | 4 | 3.1 | collagen |
| KO19-243 | 2019/1/24 | F | 261.89 | 43 | 3 | 3.6 | collagen |
| KO19-244 | 2019/1/24 | F | 202.29 | 7 | 0 | 3.9 | collagen |
| KO19-245 | 2019/1/24 | F | 262.11 | 43 | 3 | 3.9 | collagen |
| GMT19-449 | 2019/1/25 | F | 254.15 | 31 | 2 | 3.3 | collagen |
| KARU19-261 | 2019/1/27 | F | 207.04 | 7 | 0 | 2.0 | collagen |
| KO19-248 | 2019/1/28 | F | 282.72 | 43 | 3 | 3.7 | collagen |
| KO19-249 | 2019/1/28 | F | 220.82 | 7 | 0 | 4.4 | collagen |
| GMT19-457 | 2019/1/29 | F | 256.48 | 151 | 12 | 2.6 | collagen |
| GMT19-458 | 2019/1/29 | F | 276.36 | 67 | 5 | 3.4 | collagen |
| GMT19-459 | 2019/1/29 | F | 258.91 | 43 | 3 | 6.0 | collagen |
| GMT19-464 | 2019/1/30 | F | 244.66 | 31 | 2 | 2.9 | collagen |
| GMT19-465 | 2019/1/31 | F | 215.23 | 7 | 0 | 4.1 | collagen |
| KO19-253 | 2019/1/31 | F | 238.05 | 19 | 1 | 2.8 | collagen |
| KARU19-264 | 2019/2/1 | F | 266.6 | 80 | 6 | 3.9 | collagen |
| KO19-256 | 2019/2/1 | F | 219.99 | 8 | 0 | 4.3 | collagen |
| GMT19-486 | 2019/2/4 | F | 268.34 | 44 | 3 | 3.4 | collagen |
| KARU19-266 | 2019/2/5 | F | 275.57 | 68 | 5 | 1.9 | collagen |
| KARU19-267 | 2019/2/5 | F | 260.46 | 32 | 2 | 0.9 | collagen |
| GMT19-487 | 2019/2/7 | F | 261.32 | 92 | 7 | 3.3 | collagen |
| GMT19-488 | 2019/2/8 | F | 270.88 | 68 | 5 | 3.2 | collagen |
| GMT19-492 | 2019/2/15 | F | 247.64 | 32 | 2 | 1.6 | collagen |
| KARU19-269 | 2019/2/15 | F | 227.35 | 8 | 0 | 3.4 | collagen |
| KO19-259 | 2019/2/15 | F | 273.87 | 32 | 2 | 6.5 | collagen |
| GMT19-504 | 2019/2/18 | F | 217.38 | 8 | 0 | 2.8 | collagen |
| KO19-260 | 2019/2/18 | F | 239.93 | 20 | 1 | 4.0 | collagen |
| GMT19-506 | 2019/2/20 | F | 265.22 | 116 | 9 | 4.4 | collagen |
| GMT19-507 | 2019/2/20 | F | 258.12 | 44 | 3 | 3.1 | collagen |
| KARUKAN003 | 2019/2/20 | F | 275.07 | 104 | 8 | 0.0 | collagen |
| GMT19-508 | 2019/2/21 | F | 215.64 | 8 | 0 | 3.0 | collagen |
| GMT19-509 | 2019/2/21 | F | 260.72 | 68 | 5 | 2.5 | collagen |
| KO19-265 | 2019/3/3 | F | 272.35 | 81 | 6 | 3.6 | collagen |
| GMT20-1222 | 2020/1/9 | F | 234.59 | 19 | 1 | 1.8 | collagen |
| KARU20-246 | 2020/1/15 | F | 214.97 | 7 | 0 | 1.1 | collagen |
| GMT20-1237 | 2020/1/17 | F | 246.01 | 19 | 1 | 4.8 | collagen |
| KARU20-251 | 2020/1/21 | F | 212.49 | 7 | 0 | 2.2 | collagen |
| GMT20-1310 | 2020/2/3 | F | 254.91 | 68 | 5 | 1.4 | collagen |
| KARU20-259 | 2020/2/5 | F | 254.19 | 20 | 1 | 2.5 | collagen |
| KARUKAN20-002 | 2020/2/5 | F | 254.18 | 20 | 1 | 1.0 | collagen |
| KARUKAN20-005 | 2020/2/12 | F | 265.35 | 44 | 3 | 2.7 | collagen |
| GMT20-1332 | 2020/2/13 | F | 271.67 | 224 | 18 | 4.2 | collagen |
| KARU20-262 | 2020/2/14 | F | 220.95 | 8 | 0 | 3.6 | collagen |
| GMT20-1339 | 2020/2/15 | F | 255.45 | 56 | 4 | 2.2 | collagen |
| KARUKAN20-007 | 2020/2/19 | F | 242.04 | 20 | 1 | 2.3 | collagen |
| KARUKAN20-009 | 2020/2/19 | F | 275.15 | 80 | 6 | 3.5 | collagen |
| GMT20-1393 | 2020/3/3 | F | 209.12 | 9 | 0 | 2.4 | collagen |
| GMT20-1394 | 2020/3/3 | F | 209.3 | 9 | 0 | 3.0 | collagen |
| KARU20-271 | 2020/3/17 | F | 275.65 | 93 | 7 | 3.6 | collagen |
| KO20-236 | 2020/3/22 | F | 212.6 | 9 | 0 | 4.3 | collagen |
| KARU20-277 | 2020/3/25 | F | 199.25 | 9 | 0 | 3.6 | collagen |
| KO20-247 | 2020/3/31 | F | 277.71 | 45 | 3 | 4.1 | collagen |
| KO20-006 | 2020/4/13 | F | 267.49 | 58 | 4 | 2.7 | collagen |
| KARU20-010 | 2020/4/15 | F | 261.24 | 82 | 6 | 3.2 | collagen |
| KARU20-016 | 2020/4/17 | F | 268.62 | 70 | 5 | 3.1 | collagen |
| KARU20-039 | 2020/4/29 | F | 245.67 | 22 | 1 | 3.8 | collagen |
| SA23-720 | 2023/7/1 | F | 221.41 | 13 | 1 | 3.3 | bulk |
| SA23-725 | 2023/7/2 | F | 259.06 | 25 | 2 | 2.1 | bulk |
| UE23-069 | 2023/7/2 | F | 242.73 | 25 | 2 | 1.4 | bulk |
| SA23-732 | 2023/7/3 | F | 248.68 | 13 | 1 | 2.9 | collagen |
| SA23-734 | 2023/7/3 | F | 255.55 | 157 | 13 | 4.4 | collagen |
| UE23-071 | 2023/7/3 | F | 257.45 | 25 | 2 | 2.4 | bulk |
| SA23-736 | 2023/7/4 | F | 259.41 | 49 | 4 | 3.9 | collagen |
| SA23-738 | 2023/7/4 | F | 223.13 | 13 | 1 | 3.3 | bulk |
| SA23-739 | 2023/7/4 | F | 230.71 | 13 | 1 | 2.3 | bulk |
| SA23-740 | 2023/7/4 | F | 218.9 | 13 | 1 | 3.0 | bulk |
| KO23-106 | 2023/7/5 | F | 262.07 | 25 | 2 | 5.4 | bulk |
| SA23-747 | 2023/7/5 | F | 226.15 | 13 | 1 | 3.3 | bulk |
| UE23-073 | 2023/7/5 | F | 260.44 | 25 | 2 | 3.2 | bulk |
| UE23-074 | 2023/7/5 | F | 226.65 | 13 | 1 | 2.4 | bulk |
| SA23-749 | 2023/7/6 | F | 273.19 | 181 | 15 | 2.5 | bulk |
| SA23-750 | 2023/7/7 | F | 249.68 | 25 | 2 | 4.3 | bulk |
| SA23-751 | 2023/7/7 | F | 262.57 | 85 | 7 | 2.5 | bulk |
| SA23-755 | 2023/7/7 | F | 240.97 | 37 | 3 | 3.7 | bulk |
| SA23-756 | 2023/7/7 | F | 227.85 | 13 | 1 | 4.5 | bulk |
| UE23-076 | 2023/7/7 | F | 236.04 | 13 | 1 | 3.3 | bulk |
| UE23-077 | 2023/7/7 | F | 226.5 | 13 | 1 | 2.6 | bulk |
| SA23-762 | 2023/7/9 | F | 233.81 | 13 | 1 | 3.0 | bulk |
| SA23-767 | 2023/7/10 | F | 232.59 | 13 | 1 | 4.0 | collagen |
| SA23-768 | 2023/7/10 | F | 239.17 | 25 | 2 | 4.4 | collagen |
| UE23-084 | 2023/7/10 | F | 253.88 | 25 | 2 | 5.5 | bulk |
| KARU23-056 | 2023/7/11 | F | 230.75 | 13 | 1 | 3.7 | collagen |
| SA23-772 | 2023/7/11 | F | 220.51 | 13 | 1 | 3.2 | bulk |
| TATE23-005 | 2023/7/11 | F | 264.32 | 169 | 14 | 7.5 | bulk |
| UE23-085 | 2023/7/11 | F | 262.9 | 85 | 7 | 4.0 | bulk |
| SA23-775 | 2023/7/12 | F | 224 | 13 | 1 | 2.3 | bulk |
| SA23-778 | 2023/7/12 | F | 233.52 | 13 | 1 | 3.6 | bulk |
| SA23-779 | 2023/7/12 | F | 268.68 | 37 | 3 | 4.1 | bulk |
| UE23-086 | 2023/7/12 | F | 270.14 | 121 | 10 | 4.2 | bulk |
| UE23-087 | 2023/7/12 | F | 271.34 | 37 | 3 | 2.1 | bulk |
| SA23-782 | 2023/7/14 | F | 237.04 | 13 | 1 | 3.3 | bulk |
| SA23-783 | 2023/7/14 | F | 233.47 | 13 | 1 | 2.9 | bulk |
| SA23-784 | 2023/7/15 | F | 272.27 | 97 | 8 | 2.4 | bulk |
| SA23-785 | 2023/7/15 | F | 249.47 | 25 | 2 | 3.7 | bulk |
| KARU23-061 | 2023/7/16 | F | 262.17 | 25 | 2 | 4.9 | collagen |
| SA23-788 | 2023/7/16 | F | 222.79 | 13 | 1 | 1.5 | bulk |
| KARU23-062 | 2023/7/17 | F | 252.79 | 25 | 2 | 3.7 | bulk |
| SA23-790 | 2023/7/17 | F | 238.18 | 25 | 2 | 1.1 | bulk |
| SA23-791 | 2023/7/17 | F | 264.69 | 193 | 16 | 3.9 | collagen |
| SA23-792 | 2023/7/18 | F | 262.26 | 97 | 8 | 4.2 | collagen |
| GMT20-1221 | 2020/1/9 | M | 274.41 | 19 | 1 | 3.8 | collagen |
| KARU20-245 | 2020/1/13 | M | 207.55 | 7 | 0 | 0.9 | collagen |
| KARU20-244 | 2020/1/13 | M | 255.22 | 19 | 1 | 2.4 | collagen |
| GMT20-1256 | 2020/1/22 | M | 283.28 | 151 | 12 | 1.8 | collagen |
| KARUKAN20-001 | 2020/2/5 | M | 285.11 | 104 | 8 | 1.6 | collagen |
| KARUKAN20-003 | 2020/2/6 | M | 289.39 | 68 | 5 | 0.2 | collagen |
| GMT20-1315 | 2020/2/6 | M | 251.79 | 20 | 1 | 2.1 | collagen |
| KARU20-260 | 2020/2/9 | M | 255.28 | 20 | 1 | 3.1 | collagen |
| KARU20-261 | 2020/2/9 | M | 226.02 | 8 | 0 | 3.4 | collagen |
| KARUKAN20-004 | 2020/2/12 | M | 297.88 | 140 | 11 | 0.5 | collagen |
| KARU20-264 | 2020/2/21 | M | 283.42 | 44 | 3 | 1.9 | collagen |
| UE20-011 | 2020/2/21 | M | 250.38 | 20 | 1 | 3.2 | collagen |
| UE20-012 | 2020/2/26 | M | 263.66 | 20 | 1 | 2.8 | collagen |
| UE20-018 | 2020/3/5 | M | 261.4 | 21 | 1 | 2.5 | collagen |
| UE20-019 | 2020/3/5 | M | 273.06 | 33 | 2 | 3.1 | collagen |
| UE20-020 | 2020/3/6 | M | 280.5 | 33 | 2 | 2.7 | collagen |
| GMT20-1407 | 2020/3/14 | M | 258.3 | 21 | 1 | 1.8 | collagen |
| KARU20-273 | 2020/3/19 | M | 218.78 | 9 | 0 | 1.7 | collagen |
| GMT20-1414 | 2020/3/19 | M | 262.2 | 33 | 2 | 2.9 | collagen |
| KO20-235 | 2020/3/21 | M | 254.78 | 21 | 1 | 2.5 | collagen |
| KARU20-275 | 2020/3/21 | M | 258.96 | 21 | 1 | 2.9 | collagen |
| KARU20-279 | 2020/3/26 | M | 221.02 | 9 | 0 | 3.0 | collagen |
| KO20-246 | 2020/3/31 | M | 277.75 | 33 | 2 | 4.6 | collagen |
| KARU20-002 | 2020/4/5 | M | 222.33 | 10 | 0 | 1.3 | collagen |
| KARU20-028 | 2020/4/21 | M | 262.24 | 34 | 2 | 0.2 | collagen |
| KO20-013 | 2020/4/21 | M | 241.54 | 22 | 1 | 1.2 | collagen |
| KO20-012 | 2020/4/21 | M | 266.01 | 22 | 1 | 3.4 | collagen |
| KARU20-029 | 2020/4/22 | M | 261.38 | 34 | 2 | 2.1 | collagen |
| KO20-014 | 2020/4/22 | M | 230.57 | 10 | 0 | 4.1 | collagen |
| KARU20-038 | 2020/4/29 | M | 236.08 | 10 | 0 | 0.5 | collagen |
| SA23-721 | 2023/7/1 | M | 236.71 | 13 | 1 | 3.1 | bulk |
| KARU23-054 | 2023/7/1 | M | 267.42 | 25 | 2 | 3.9 | collagen |
| SA23-719 | 2023/7/1 | M | 242.39 | 13 | 1 | 4.7 | bulk |
| MI23-002 | 2023/7/2 | M | 266.37 | 25 | 2 | 2.1 | bulk |
| SA23-724 | 2023/7/2 | M | 246.43 | 25 | 2 | 2.6 | bulk |
| SA23-726 | 2023/7/2 | M | 296.1 | 121 | 10 | 3.2 | bulk |
| UE23-067 | 2023/7/2 | M | 245.17 | 13 | 1 | 3.4 | bulk |
| UE23-068 | 2023/7/2 | M | 243.92 | 13 | 1 | 3.4 | bulk |
| SA23-722 | 2023/7/2 | M | 280.07 | 109 | 9 | 4.1 | bulk |
| KO23-101 | 2023/7/3 | M | 249.75 | 13 | 1 | 4.1 | bulk |
| SA23-737 | 2023/7/4 | M | 247.6 | 13 | 1 | 2.9 | bulk |
| KO23-103 | 2023/7/4 | M | 265.18 | 25 | 2 | 3.1 | bulk |
| SA23-741 | 2023/7/4 | M | 227.66 | 13 | 1 | 5.2 | bulk |
| KO23-105 | 2023/7/5 | M | 237.94 | 13 | 1 | 0.3 | bulk |
| UE23-072 | 2023/7/5 | M | 237.32 | 13 | 1 | 2.1 | bulk |
| SA23-743 | 2023/7/5 | M | 262.77 | 25 | 2 | 2.8 | bulk |
| SA23-754 | 2023/7/7 | M | 232.9 | 13 | 1 | 2.2 | bulk |
| UE23-075 | 2023/7/7 | M | 277.26 | 25 | 2 | 3.8 | bulk |
| UE23-079 | 2023/7/8 | M | 239.44 | 13 | 1 | 1.9 | bulk |
| SA23-758 | 2023/7/8 | M | 229.79 | 13 | 1 | 2.5 | collagen |
| SA23-757 | 2023/7/8 | M | 237.35 | 13 | 1 | 3.5 | bulk |
| SA23-759 | 2023/7/8 | M | 252.17 | 13 | 1 | 4.1 | collagen |
| UE23-078 | 2023/7/8 | M | 266.04 | 25 | 2 | 4.5 | bulk |
| SA23-760 | 2023/7/8 | M | 260.12 | 25 | 2 | 5.3 | bulk |
| UE23-081 | 2023/7/9 | M | 277.51 | 25 | 2 | 1.7 | bulk |
| UE23-080 | 2023/7/9 | M | 244.77 | 13 | 1 | 1.9 | bulk |
| SA23-761 | 2023/7/9 | M | 230.12 | 13 | 1 | 4.4 | collagen |
| SA23-764 | 2023/7/10 | M | 240.46 | 13 | 1 | 2.6 | bulk |
| SA23-765 | 2023/7/10 | M | 233.98 | 13 | 1 | 2.8 | bulk |
| SA23-763 | 2023/7/10 | M | 251.93 | 13 | 1 | 3.1 | bulk |
| SA23-766 | 2023/7/10 | M | 247.16 | 13 | 1 | 5.2 | bulk |
| SA23-770 | 2023/7/11 | M | 224.22 | 13 | 1 | 3.6 | bulk |
| SA23-769 | 2023/7/11 | M | 226.91 | 13 | 1 | 3.8 | bulk |
| SA23-776 | 2023/7/12 | M | 265.08 | 25 | 2 | 2.3 | bulk |
| UE23-088 | 2023/7/13 | M | 264.74 | 25 | 2 | 2.9 | bulk |
| SA23-780 | 2023/7/13 | M | 267.56 | 25 | 2 | 3.1 | bulk |
| KO23-114 | 2023/7/14 | M | 243.82 | 13 | 1 | 3.1 | bulk |
| KARU23-060 | 2023/7/14 | M | 249.16 | 13 | 1 | 3.2 | bulk |
| KARU23-059 | 2023/7/14 | M | 257.01 | 25 | 2 | 3.8 | bulk |
| SA23-787 | 2023/7/16 | M | 285.45 | 49 | 4 | 1.6 | bulk |
| KO23-115 | 2023/7/16 | M | 272.85 | 25 | 2 | 1.6 | bulk |
| KARU23-063 | 2023/7/17 | M | 264.39 | 25 | 2 | 2.6 | collagen |
| SA23-796 | 2023/7/20 | M | 233.37 | 25 | 2 | 1.5 | bulk |
| KO23-118 | 2023/7/20 | M | 252.75 | 13 | 1 | 2.1 | bulk |
|  |  |  |  |  |  |  |  |
