# Supplementary material for: Silver-spoon effect in agricultural crop consumers: crop consumption enhances skeletal growth in sika deer
Source: PeerJ. 2025 Aug 7;13:e19836. doi: 10.7717/peerj.19836 (PMC12335832; doi:10.7717/peerj.19836)
Supplement: Supplemental Information 4 — The δ15N values were measured for bone collagen samples of mother. [file peerj-13-19836-s004.docx]

| ID | hunting date | sex | Hind-foot length (cm) | estimated gestation period (day) | age of mother | δ^15^N of mother (‰) |
| --- | --- | --- | --- | --- | --- | --- |
| GMT20-1237F | 2020/1/17 | F | 2.8 | 85 | 1 | 4.8 |
| GMT20-1310F | 2020/2/3 | F | 5.5 | 123 | 5 | 1.4 |
| KARU20-259F | 2020/2/5 | F | 4.5 | 105 | 1 | 2.5 |
| GMT106F | 2018/2/5 | F | 6 | 117 | 9 | 2.8 |
| KO221F | 2018/2/7 | F | 6 | 129 | 8 | 3.6 |
| GMT118F | 2018/2/12 | F | 7 | 133 | 12 | 2.7 |
| GMT123F | 2018/2/20 | F | 7.5 | 138 | 13 | 1.3 |
| KARU20-269F | 2020/3/13 | F | 12 | 173 | 5 | 6.0 |
| KO236F | 2018/3/31 | F | 13.5 | 195 | 9 | 3.5 |
| KARU20-010F | 2020/4/15 | F | 15 | 201 | 6 | 3.2 |
| KO013F | 2018/4/15 | F | 14.5 | 191 | 1 | 3.5 |
| KARU20-016F | 2020/4/17 | F | 16 | 212 | 5 | 3.1 |
| KARU20-015F | 2020/4/17 | F | 14 | 194 | 2 | 3.5 |
| KARU003F | 2018/4/22 | F | 14.5 | 198 | 5 | 0.7 |
| KARU007F | 2018/5/3 | F | 19 | 232 | 5 | 1.7 |
| KO031F | 2018/5/15 | F | 17 | 215 | 2 | 4.0 |
| KARU20-241F | 2020/1/6 | M | 3.5 | 96 | 8 | 2.7 |
| GMT060F | 2018/1/12 | M | 3 | 82 | 3 | 2.0 |
| KO20-245F | 2020/1/17 | M | 15 | 199 | 11 | 5.0 |
| GMT075F | 2018/1/23 | M | 4.6 | 103 | 9 | 2.4 |
| GMT078F | 2018/1/26 | M | 4.8 | 110 | 8 | 2.9 |
| KO0127F | 2018/1/27 | M | 4.8 | 110 | 7 | 3.9 |
| GMT088F | 2018/1/28 | M | 5.5 | 119 | 9 | 2.5 |
| KARUKAN20-002F | 2020/2/5 | M | 4 | 100 | 1 | 1.0 |
| KO219F | 2018/2/5 | M | 3.8 | 94 | 4 | 4.3 |
| KO220F | 2018/2/7 | M | 5.5 | 110 | 1 | 3.4 |
| KO222F | 2018/2/9 | M | 6.5 | 130 | 4 | 2.9 |
| GMT112F | 2018/2/11 | M | 5 | 109 | 2 | 3.3 |
| KARUKAN20-005F | 2020/2/12 | M | 7 | 130 | 3 | 2.7 |
| GMT20-1339F | 2020/2/15 | M | 6.5 | 134 | 4 | 2.2 |
| KARUKAN20-007F | 2020/2/19 | M | 4 | 102 | 1 | 2.3 |
| KARUKAN20-009F | 2020/2/19 | M | 8 | 148 | 6 | 3.5 |
| GMT148F | 2018/2/26 | M | 4 | 92 | 2 | 2.3 |
| SA008F | 2018/3/3 | M | 7.5 | 142 | 9 | 4.0 |
| KARU194F | 2018/3/31 | M | 9.5 | 169 | 1 | 0.1 |
| KO20-247F | 2020/3/31 | M | 15 | 203 | 3 | 4.1 |
| KO20-006F | 2018/4/6 | M | 17 | 218 | 4 | 2.7 |
| KO006F | 2018/4/6 | M | 15 | 203 | 6 | 6.0 |
| KARU004F | 2018/4/25 | M | 15.3 | 204 | 4 | 3.1 |
| KARU015F | 2018/5/14 | M | 19 | 233 | 2 | 2.9 |
| KARU014F | 2018/5/14 | M | 8.5 | 152 | 7 | 3.4 |
| KARU023F | 2018/5/17 | M | 16 | 210 | 8 | -0.5 |
|  |  |  |  |  |  |  |
