# Supplementary material for: Silver-spoon effect in agricultural crop consumers: crop consumption enhances skeletal growth in sika deer
Source: PeerJ. 2025 Aug 7;13:e19836. doi: 10.7717/peerj.19836 (PMC12335832; doi:10.7717/peerj.19836)
Supplement: Supplemental Information 5 — Several food samples were added on the list which reported in previous study (Hat et al. 2021). Food samples were collected from 2017 to 2024 in the study area. [file peerj-13-19836-s005.docx]

| Category | Food item | Species | n | δ15N(‰) mean ± SD |
| --- | --- | --- | --- | --- |
| Agricultural crops |  |  | **30** | **2.3 ± 2.8** |
|  | Pasture grasses |  | 22 | 2.4 ± 1.7 |
|  |  | *Dactylis glomerata* | 2 | 2.8 ± 0.2 |
|  |  | *Phleum pratense* | 5 | 2.0 ± 0.8 |
|  |  | *Lolium perenne* L. | 4 | 3.6 ± 1.1 |
|  |  | *Phalaris arundinacea* | 3 | 3.4 ± 0.7 |
|  |  | *Poa pratensis* L. | 5 | 2.8 ± 1.4 |
|  |  | *Agrostisalba* L. | 3 | -0.2 ± 2.3 |
|  |  |  |  |  |
|  | Vegetables |  | 8 | 2.0 ± 4.7 |
|  |  | *Brassica rapa var. pekinensis* | 2 | 0.2 ± 0.9 |
|  |  | *Brassica oleracea var. capitata* | 5 | 0.5 ± 2.2 |
|  |  | *Brassica oleracea var. italica* | 1 | 13.3 |
|  |  |  |  |  |
| Wid plants |  |  | **51** | **-6.2 ± 3.5** |
|  | Woody plants |  | 28 | -7.9 ± 2.6 |
|  |  | *Clethra barbinervis* | 7 | -7.1 ± 2.1 |
|  |  | *Viburnum* sp. | 8 | -8.6 ± 2.5 |
|  |  | *Acer rufinerve* | 5 | -10.9 ± 0.5 |
|  |  | *Euonymus sieboldianus var. sieboldianus* | 4 | -6.7 ± 0.8 |
|  |  | *Sorbus commixta* | 4 | -8.2 ± 1.2 |
|  |  |  |  |  |
|  | Herbs |  | 21 | -3.6 ± 3.0 |
|  |  | *Sasa* sp. | 17 | -4.0 ± 2.7 |
|  |  | *Reynoutria japonica* | 1 | 3.2 |
|  |  | *Vicia unijuga* | 1 | -1.8 |
|  |  | *Boehmeria spicata* | 1 | -6.9 |
|  |  | *Rubus palmatus* | 1 | -2.4 |
|  |  |  |  |  |
|  | Nuts |  | 2 | -2.7 ± 0.5 |
|  |  | *Quercus crispula* | 2 | -2.7 ± 0.5 |
|  |  |  |  |  |
| Other | Bait |  | 1 | 0.2 |
|  |  | Hay cube | 1 | 0.2 |
|  |  |  |  |  |
|  |  |  |  |  |
